# A virtual deliberative public engagement study on heritable genome editing among South Africans: Study protocol

**DOI:** 10.1371/journal.pone.0256097

**Published:** 2021-08-19

**Authors:** Donrich Thaldar, Beverley Townsend, Marietjie Botes, Bonginkosi Shozi, Siddharthiya Pillay

**Affiliations:** 1 School of Law, University of KwaZulu-Natal, Durban, South Africa; 2 African Health Research Flagship, University of KwaZulu-Natal, Durban, South Africa; Universitat Luzern, SWITZERLAND

## Abstract

This article outlines the protocol for a prospective study for virtual deliberative public engagement on heritable genome editing in humans. The study intends to create a platform for a diverse group of 25–30 South Africans to engage with a facilitator and each other on 15 policy questions regarding heritable genome editing, with a focus on: a) the prevention of heritable genetic conditions; b) editing for immunity; and c) editing for enhancement. The aim is to understand the views on these issues so as to inform further research and policy, and to analyse the process and effect of deliberation on opinion. Participants will be expected to study the provided resource materials and pass the entrance exam—aligning with the protocols of the Harvard Personal Genome Project. In this way, the commitment, openness and basic knowledge of the candidates will be tested to ascertain whether they are suitable participants for the deliberative engagement.

## I. Introduction

Following the actions of a rogue scientist in China in 2018 regarding the now infamous case of human heritable genome editing, scientists and ethicists called for a global moratorium on the clinical uses of this kind of genome editing until an international framework is established [1]. Included among these experts were pioneers of the CRISPR-Cas9 technology itself, such as Dr Feng Zhang and Dr Emanuelle Charpentier. This call is not without contestation, and the world of science and its related ethics now deliberate extensively on this topic [2]. Yet, missing from these deliberations is the critical and dynamic voice and consensus of lay society [3].

The International Commission on the Clinical Use of Human Germline Genome Editing recently issued an appeal for ‘extensive societal dialogue’ on the topic of human germline genome editing, recognising the political, ethical and pragmatic dimensions the public add to the success and development of new technologies [4]. How the dialogue is conducted is a key consideration—for highly technical developments like genome editing, which pose complicated political, social and ethical questions, it is not enough to conduct polls or ask opinions without creating an environment for collective reasoning and reflection. This invites the use of deliberative engagement wherein experts mediate face-to-face deliberation with a group of participants, enabling ‘long-held views of a pluralistic society [to] be revised through contestatory civic engagement’ [3].

The protocol outline in this article guides the study in which we seek to create a platform for deliberative engagement on the topic of human heritable genome editing for various purposes. This aims to understand the views of the public, and to create a platform that allows for knowledge dissemination, reflection and collective reasoning. As a meta-analysis, we also seek to understand the permanency of change as a result of the deliberations—i.e. should a participant change his or her opinion on a certain issue owing to the deliberations, how quickly does this occur, and do they revert to their initial opinion in a short period of time. Due to the current pandemic, this protocol is designed for virtual engagement. As this is still an emerging norm of engagement in academic studies, this protocol will contribute significantly to research design for virtual engagement.

## II. Review of current literature on deliberative public engagement

### i. Theoretical foundations

Involving the public in issues of policymaking through engagement has increasingly gained support [5]. John Rawls and Jürgen Habermas are key progenitors in modern deliberative democracy, a form of democracy in which deliberation is a central component in the process of decision-making [6]. Rawls and Habermas describe and explore notions involving the concepts of ‘public participation’ in questions of ‘political policy’ to establish ‘just normative social forms’ by means of ‘deliberation’.

By illustration, a fundamental aspect of Rawlesian ‘public reason’ is that a plurality of conflicting, reasonable, and comprehensive doctrines within society may be attributable to a ‘culture of free institutions’—one in which citizens are allowed to consider the reasons they give to one another when ‘fundamental political questions are at stake’ [7–10]. Similarly, Habermas in *Between Facts and Norms* elucidates the pivotal role public discourse plays in democracy and offers a bridge between normative (or standards-related frameworks) and empirical (that which is verifiable by observation or experience) approaches to democracy [11].

Habermas’s work is frequently cited as the theoretical foundation for establishing normative rationales for deliberation [12]. His version of ‘discourse ethics’ presupposes justice as a basis upon which the discursive conditions for valid, social norms are formed—this with the intention of guiding the moral reasoning of such discourse. The underlying principle of discourse speaks of ‘valid norms’ as those that have been approved by all affected individuals, concluded on the basis of democratic, fair processes of public debate and informed decision-making. Drawing on Habermas’s theory of communicative action—which establishes cooperative action undertaken by individuals based on mutual deliberation and argumentation—we can seek to understand and construct the concepts of, and tensions inherent in, the duality of modern policy making [13]. We understand that interactions are woven together, and life structured, through a process of mutual understandings, informed by conditions that both enable and limit them. This process serves not simply to aggregate preferences, but to engage in ‘thoughtful interaction and opinion formation’, so enabling people to become informed of the better arguments which in turn may influence their preferences [11].

Integrating the Rawlesian notion of public reason [10], and Habermas’s arguments in favour of discourse ethics through deliberation, public deliberation broadens democratic practice through a process of weighing up different options [14]. This in an attempt to understand the public’s view of various aspects and applications of novel technologies. It does this by raising awareness, addressing societal concerns, seeking to promote better understanding of the nuanced complexities involved, and ultimately in finding legitimate and sustainable policy solutions [15, 16].

### ii. The role of public engagement in the discourse on novel technologies such as genome editing

Scientific research and innovation do not occur within a socio-normative vacuum. Technological advancements, particularly those that proceed rapidly, are sometimes contentious, and have far-reaching effects that require input from wider society. Public engagement mechanisms have been applied in various areas of biotechnology to address concerns [17–21]. This is premised on the belief that members of society hold local knowledge and insight that may prove valuable in discussions on issues of biotechnological policy-making [22].

A 2017 report on *Human Genome Editing*: *Science*, *Ethics*, *and Governance* sets out recommendations with regard to public engagement in genome editing governance [23]. The report emphasises the role of public education and engagement in assessing and applying societal values to the risks and benefits of genome editing technologies. It refers to the need for ‘transparent and inclusive public policy debates’ before, for instance, any authorisation for clinical trials that may have a non-therapeutic purpose. In addition to risk assessment and stringent oversight mechanisms, the report indicates ‘broad participation and input by the public’ [23].

The need for new democratic approaches to policy development and governance are reiterated by various scholars. Abelson et al advance the requirement for interaction between decision-makers and the public, and for deliberation among more informed participants in complex decision-making [14]. This process involves the weighing of the evidence on issues, discussion and debate on various potential decision options, and arriving at mutually agreed upon decisions [14]. Baylis suggests a basis of ‘broad consensus’ achieved through ‘broad-based participation by persons from around the world with a range of perspectives and interests’ [24, 25]. Similarly, Jasanoff et al advocate that ‘good governance depends on visions of progress that are collectively defined, drawing on the full richness of the democratic imagination.’ [26]. They caution that dismissing public views ‘deprives society of the freedom to decide what forms of progress are culturally and morally acceptable’ [26]. Cavaliere et al adopt what they term an ‘enlightened democracy approach’ [27]. This presents a position that combines the adherence of democratic values within well-reasoned and informed decision-making, thereby seeking to adopt an approach of greater consolidation—rather than one of fragmentation—and one that is inclusive of the differing views of experts [27]. Burgess likewise demonstrates that it is possible to converge policy-making responsibility with the input of the public in an attempt to ‘co-produce’ policy and standards of practice on matters of a technical nature [22]. How, then, do we go about obtaining such participatory public input?

### iii. The process of public engagement: A review of the literature

Broadly, deliberative public engagement is an attempt to answer the question: what do people think should be done within a given context, if they are given the opportunity to consider fundamental issues regarding a certain subject under favourable conditions [6]? Furthermore, deliberative public engagement also serves to inform and educate the public; to consolidate public values, assumptions, and preferences; to strengthen the quality of decisions through deliberation; and to foster trust and reduce conflict in effective decision-making [12].

#### a) Decisions about representation and sample size

The sample should include a small, odd number of participants, ideally 13 to 25 participants, with Whitty et al suggesting that sample sizes contain a minimum of 20 participants [28, 29]. The participants should be a small selection of diverse people contained in a stratified sample and should not necessarily be representative of a community or a region as a whole. Although a sample of this quantity cannot be statistically representative of the population of the region, the aim is to attain diversity, minimise selection bias, and maximise the range of views obtained [29]. As the point of the process is to advance arguments from all sides of a debate, sample sizes that are too small should be avoided [6]. Diverse samples, albeit small ones, can nevertheless add significant perspectives to policy-making processes, as research by Goodin and Dryzek shows that ‘mini-publics’ can influence ‘macro-politics’ [30].

#### b) Recruitment strategy and design

Demographics including ethnicity, gender, education, and income are frequently used. The proposition is that participants selected from a variety of demographic backgrounds will increase the likelihood and prevalence of divergent views and attitudes [6]. Attitudinal representativeness, that is, to include participants of different attitudes, is important [6]. The over-representation of certain groups, particularly those with life experiences relevant to the topic, can provide specific insights [6, 31].

The literature suggests that participants selected should be those who are responsive to alternative views, are amenable to changing their opinions, do not have extreme positions, can formulate their reasons for adopting a certain viewpoint, and are willing to engage and participate in communicative processes [32, 33]. Setting out clear objectives in the design phase, confronting assumptions that might frame the discussions, and identifying key values that we hope to explore, we provide an overall structure for the debate—one that enables discourse but avoids manipulation [33]. Respectful deliberations foster a climate where participants are comfortable to share views, challenge experts’ opinions, and actively participate without fear of reprisal [33].

#### c) Providing information and the selection of ‘experts’

The neutral and objective provision of information and material is central to the deliberations. Statements of fact should be differentiated from the values, judgments and opinions of the experts [34].

For the deliberation to be of value, the participants need not be proficient or skilled in the subject, but should be sufficiently familiar and knowledgeable about the various issues, so as to enable them to contribute meaningfully and insightfully to the discussions [35].

#### d) Facilitation

During deliberation, an attempt is made to prevent the discursive dominance of individual participants. The apprehension is that the process may be hijacked to promote a certain agenda and/or be biased to favour one particular perspective [31]. Unduly influencing or framing the debate is a risk [36].

#### e) The structure of the deliberation process

Deliberation turns on the notion of ‘weighing’, that is, the evaluating and balancing of various competing arguments [6]. The participants should be given the opportunity to engage in discussions for and against various positions, in order to gain knowledge and potentially change their opinions [6]. As the process of deliberation unfolds the participants become more informed, acknowledge their views and those of others, and realise the implications of such views with respect to the issues [6]. Reactions to genome editing applications, for instance, may be influenced by the apprehension over ‘playing God’, the fear of eugenics, or the creation of ‘designer babies’—all views that are deeply influenced by religious, cultural, and historical roots in societies [37, 38].

As suggested by Burall, the public is not an ‘undifferentiated mass’ [16]. Rather than a set of unchanging and rigid perspectives awaiting ‘identification’ and ‘discovery’, the design should be accommodating of a broad range of equally valid and plausible perspectives. Deliberation allows arguments to be advanced and facilitated with the opportunity of the participants to add further arguments and perspectives. This encourages nuanced conversations [16]. The authenticity of the deliberation depends on the ability of participants to reflect on differing views, entertain various perspectives and ultimately establish preferences in a non-coercive manner [5]. Fishkins suggests that the process requires experts with competing viewpoints—as when participants realise that the experts disagree, they may be more inclined to assess the competing positions, rather than merely defer to a particular expert [6]. Experts can answer questions from the participants, but the experts should be discouraged from participating in the deliberation, as this is an attempt to obtain the public’s views without undue influence [39].

#### f) Outcomes and decisions arising from the process

The participants should be encouraged to reach their own conclusions. The change in a participant’s opinion is not a necessary outcome of the process and is not an indicator of a successful deliberation [6]. Two design aspects worthy of caution that can cause distortions are that of: (1) the more advantaged participants within the group dominating discussions and thereby imposing their views on the others, causing distortions in the outcome; and (2) that of polarisation where a participant holding a particular opinion before the deliberation, moves, after deliberation, to a position that strengthens their existing opinion, thus moving them toward a more extreme point in the direction of their own pre-deliberation opinions. Sunstein describes an example of polarisation where people who are opposed to, for instance, gun control, who after discussions with other members of the group, become even more opposed to gun control [32]. To prevent distortions, the process requires taking cognisance of, and adopting measures, to reduce domination by certain participants and to minimise the occurrence of participants moving to more extreme positions.

Opinions should be measured and documented before and after deliberation [32]. Using confidential questionnaires can reduce the effect of social comparison [6]. Consensus in this regard is defined by Dryzek as the unanimous agreement on the course of action and the reasons for it [5]. Arriving at consensus in a pluralistic world is not always attainable nor desirable [5]. The social pressure to reach a unanimous decision should thus be avoided [6]. Rather, we should strive to achieve a workable agreement on the agreed course of action, albeit for differing reasons [5]. Consensus should not be forced, and if not reached, should in no way be seen as a failure of the deliberation process.

## III. Materials and methods

This section outlines in detail the protocol that will be followed so as to ascertain public opinion on human genome editing through deliberative engagement, and to understand how deliberations affect the permananecy of a change in opinion. The protocol has received ethics clearance from the Humanities and Social Sciences Research Ethics Committee of the University of KwaZulu-Natal. (Protocol reference number: HSSREC/00002595/2021; S1 Document).

In the context of Covid-19’s physical engagement limitations, and considering that Virtual Research Environments (VRE) are gaining momentum to re-shape the research landscape at academic institutions [40], we decided to experiment by conducting our engagement study via the video communications platform ‘Zoom’. Thus, this protocol adds to the emerging methodology on *virtual* deliberative public engagement, which is becoming a norm across academic research due to the global pandemic.

### i. Participant recruitment

We intend to recruit a small collection of people of different races, genders, ages, educational backgrounds, and religious affiliations, provided they are South African citizens over the age of 18 years, and are proficient in English as a verbal and written language. As deduced from the literature review, this will comprise of 25 participants. The purpose of the study will be to conduct a small-scale deliberative engagement in the style described and adopted by Burgess [15, 22, 33], albeit online, with the aim of obtaining input from individuals who do not form part of any special interest group, and would thus otherwise be unlikely to be represented in political processes.

The recruitment will be conducted in five phases:

1. Sortition (A). Social media ads will be used to invite participation by any person residing in South Africa. These advertisements will continue until an appropriate demographic sample is selected.
2. Self-selection. Candidates can then decide whether to express interest in the project by following the link to the project website.
3. Selection (A). On the project website, interested candidates will be requested to provide informed consent for eligibility screening (S2 Document), and then proceed to complete a short questionnaire (S3 Document). The purpose of the questionnaire is to assess whether the candidate is eligible to become a participant in the study, and to gather demographic information about the candidate that the research team will use to select an inclusive group of participants. The eligibility criteria are: (1) Citizenship of South Africa, (2) being of the age of majority (18 years), (3) willingness to learn more about genome editing and the ethical debates surrounding it, (4) willingness to form one’s own opinions on the various uses of genome editing and to discuss one’s opinions on genome editing with others, (5) willingness to participate in three evenings of online meetings that will be recorded for research purposes, and (6) having sufficient internet and Zoom access. (Internet data will be provided, where required.) Only those candidates who comply with all the eligibility criteria will be allowed to register on the website. In addition, the questionnaire will also gather the following demographic information of candidates: race, gender, educational attainment, age, and religion/belief.
4. Selection (B). All registered candidates will be given access to resource material (S4 Document) and will be requested to take an online entrance exam to assess their comprehension of the resource material. The entrance exam is only passed if all its questions are answered correctly. However, there is no limit on the number of times an eligible candidate can sit the entrance exam. Only those registered candidates who pass the entrance exam will become part of a pool of potential participants. All the potential participants will be requested to record their informed consent to participate in the study (S5 Document).
5. Sortition (B). From the potential participant pool, 30 participants will be selected in such a way as to have group of participants that are as inclusive as possible (note not representative) on the metrics of demographic information mentioned above. Although the literature recommends 25 participants, in anticipation of attrition occurring we will select an additional 5 participants—bringing the total number of individuals invited to participate in the study to 30. This is aligned to an approach adopted in a study reported by O’Doherty et al [41].

### ii. Measures

The study will be structured around fifteen policy proposals related to heritable genome editing. Each proposal will start with the formula ‘Our country’s laws should allow parents to choose to use genome editing before a child’s birth to…’ In the case of each proposal, participants will have three possible responses:

- Yes, always.
- Yes, but subject to certain conditions, like that genome editing must be safe. If there are other conditions, please state.
- No, never.

In the case of each proposal, if the response is affirmative—either absolute or qualified—participants will also be asked to consider who should pay for the genome editing. The possible responses are:

- Self.
- Private medical aid.
- Government (through the new National Health Insurance).
- Other, please state.

The standard formula ‘Our country’s laws should allow parents to choose to use genome editing before a child’s birth to……’ is based on the assumption that, in the event that specific applications of heritable genome editing are allowed in an open and democratic society, like South Africa aspires to be, the decision to use it will be the parents’ decision—in other words, not the decision of inter alia the state and healthcare professionals. We suggest that this is in line with the right to reproductive autonomy, which is protected in the South African Constitution, [42] and that applies to the parents’ use of new reproductive technologies [43].

Regarding the question of payment, South Africa is in the process of moving towards a National Health Insurance that is designed to pool funds together to provide access to comprehensive healthcare services for all. The National Health Insurance Fund will cover treatments that fall within the prescribed Formulary [44]. In the event that specific—if not all—applications of heritable genome editing are listed in the Formulary, parents can use the services at accredited health facilities free of charge [44].

The fifteen policy proposals are designed to cover a broad spectrum of potential applications of heritable genome editing. Each policy proposal rests on the technical assumption that genome editing will, at some time in the future, be able to impact the trait to which it relates.

If participants object to using heritable genome editing itself, the answer to all the policy proposals will be ‘no’. However, public opinion surveys in the rest of the world have shown that people’s opinions on allowing the use of heritable genome editing are often connected to the specific application of heritable genome editing, and differ depending on the specific application [45].

The fifteen policy proposals are as follows: Our country’s laws should allow parents to choose to use genome editing before a child’s birth to–

1. prevent the child being born with a serious heritable disease like sickle cell anaemia, muscular dystrophy, or Alzheimer’s.
2. prevent the child being born with a less serious heritable disease, like asthma, or eczema.
3. prevent the child being born with a disability like deafness or blindness.
4. prevent the child being born with Down’s syndrome.
5. prevent the child being born with albinism.
6. make the child immune to contracting a serious disease like TB or HIV/Aids during the child’s life.
7. make the child immune to contracting Covid-19 during the child’s life.
8. make the child immune to contracting an illness like the flu or a common cold during the child’s life.
9. influence how intelligent the child will be.
10. influence how athletic the child will be.
11. influence how aggressive the child will be.
12. influence how cooperative the child will be.
13. influence the sexual orientation of the child.
14. determine the child’s skin tone (lighter or darker).
15. determine the child’s eye colour.

The first five policy proposals relate to the prevention of heritable genetic conditions. We start with serious heritable disease, which is often perceived as the strongest candidate for ‘justifying’ the use of heritable genome editing. We then compare serious heritable disease with heritable diseases that are less serious and can typically be managed through medical intervention without significant sacrifices to a person’s functioning in society. Next, we seek to ascertain whether our participants feel different about heritable disease vis-à-vis heritable disability such as deafness and blindness. Given the increase in the use of non-invasive prenatal testing (NIPT) to test for Down’s syndrome in particular (with the intention to terminate a pregnancy if the NIPT reveals Down’s syndrome), we decided to place Down’s syndrome on its own. Similarly, given that albinism has historically been associated with social stigma, we have also placed albinism on its own.

Policy proposals 6 to 8 relate to editing-for-immunity—a kind of genetic ‘vaccine’. For some, there is a morally significant distinction between ‘correcting’ a genetic defect that may cause heritable disease or disability—in other words restoring genetic ‘normality’—and changing a normal human genome to provide some super-normal advantage, such as immunity against infectious disease. The former kind of editing is often referred to a ‘therapeutic’, and the latter as ‘enhancement’. If this nomenclature is adopted, policy proposals 6 to 14 are all ‘enhancements’, with policy proposals 6 to 8 being health-related ‘enhancements’. We seek to ascertain whether the participants view serious disease differently from less serious disease.

One should consider that South Africa carries a heavy infectious disease burden, apart from Covid-19. For instance, the Global TB Report of 2015 estimated that in 2014 South Africa had the second highest TB incidence rate in the world, with 834 cases per 100 000 population [46]. The province where this study will be done, KwaZulu-Natal, is one of the provinces with the highest TB burdens [47]. The municipal region where this study will take place, eThekwini, had the highest number of TB cases in South Africa, and also one of the lowest treatment success rates (67.9%) [48]. Tuberculosis remained the leading cause of death in South Africa in 2014, with 8.4% of deaths nationally attributed to TB.

Given the worldwide Covid-19 pandemic, and the far-reaching government actions that were taken to manage this pandemic, such as a nationwide lockdown in South Africa, we felt it appropriate to include a policy proposal that specifically deals with Covid-19. We seek to ascertain whether the participants, after living through these drastic government measures, view immunity to Covid-19 differently from immunity to other diseases.

In policy proposals 9 to 13, the verb ‘influence’ is used to communicate that genetics may play a significant, but not the only role in determining the trait in question. Policy proposals 9 and 10 deal with the stereotypical enhancement candidates, athleticism and intelligence. We have decided to deal with them separately to ascertain whether participants view them differently. Our thinking was that while athleticism may be associated more with perceived unfair advantages because of genetic characteristics—think, for instance, of the controversy surrounding the South African Olympic athlete Castor Semenya—intelligence may be comparatively less associated with perceived unfair genetic advantage, and may also be perceived as being important for success in life.

Policy proposals 11 to 13 deal with traits (aggressiveness, cooperativeness, and sexual orientation) that may be perceived as contributing to a person’s chances of success and fitting in in our current society, but are also intertwined with ever-evolving cultural notions of preferred behaviour. Importantly, aggressiveness and cooperativeness are also traits that can be moulded, at least to an extent, through free will and practice, by persons themselves. On the other hand, most people would agree that a person’s sexual orientation is not the person’s own moral decision; however, whether to identify as having a certain sexual orientation, and live one’s life accordingly, can be perceived as a moral decision. As such, aggressiveness, cooperativeness, and sexual orientation may fall in a category of traits that can to some extent be linked to one’s own moral agency. This category of traits is important in the light of an argument presented by John Harris, namely that being able to make one’s own moral decisions—whether good or bad—is quintessential to why we value *being human*, and is therefore an ability that should not be manipulated [49].

The last two policy proposals are purely aesthetic: the child’s skin tone and eye colour. Similar to traits like intelligence, aggressiveness, and cooperativeness, skin tone and eye colour might contribute to a person’s chances of success in society. However, aesthetic judgments tend to be more subjective and culturally influenced than intelligence; and unlike aggressiveness and cooperativeness that are linked to one’s own moral agency, parents’ aesthetic judgments regarding skin tone and eye colour will not influence the child’s moral agency.

### iii. Methods

A week before the engagement sessions begin, all participants will be provided with access to a webpage with the fifteen policy questions that will be deliberated on at the engagement. Each participant will be requested to consider all the policy questions and indicate his or her answer at that (pre-deliberation) stage.

Whereas our physical engagement was planned for half a day, we decided that in the Zoom context we could more easily lose participant attention. We therefore decided to conduct the engagement in three 1½ hour sessions, over three consecutive weekday evenings to support appropriate collaborative activities, maintaining awareness [50] and maintaining multiple engagements in activities [51].

At the first evening, all participants and facilitators will introduce themselves. The plenary facilitator will establish the ground rules, such as that participants should endeavour to limit their speaking turns to 30 seconds, and 1 minute at the longest. After this, the agenda will be introduced to the participants. In particular, the participants will be provided with the policy questions that they will have to deliberate on that evening. Each evening will have a theme:

- Evening 1: prevention of heritable genetic conditions
- Evening 2: editing for immunity
- Evening 3: editing for enhancement

At each of the three evenings, after having time for questions, the plenary meeting will be broken up into five randomly selected sub-groups, each with its own facilitator. Each sub-group will have an initial 20 minutes to deliberate on the policy proposals entailed by the evening’s theme. This will be followed by an initial plenary deliberation of 20 minutes. If consensus eludes the deliberation at this point, the process of breaking up into sub-groups and returning for a plenary meeting will be repeated. Note that the sub-groups will not remain the same, but will be randomly re-composed for the second round of break-away deliberations; similarly, the sub-groups will also be randomly re-composed every evening. The aim is to attempt to avoid that the members of a sub-group become entrenched in their sub-group position.

Throughout deliberations, consensus will not be forced. If it becomes clear that there is persistent disagreement on an issue, the clear articulation and documentation of all remaining positions will be seen as a satisfactory outcome. Large group discussions on each issue will be concluded with a final vote on the recommendation(s). The vote will serve two purposes: (1) provide closure to discussion on an issue and allow the facilitator to move the discussion forward; and (2) ensure that minority views are not missed and are clearly documented.

Law academics and postgraduate law students will be trained to act as facilitators, who may be asked any questions on the topics should they arise.

A week after the engagement is done, each participant will again be requested to answer the policy questions that were deliberated on at the engagement on his or her own. The purpose will be to investigate whether individual participants changed their mind at the deliberation, and whether they may again change their minds after the (virtual) social context of the deliberation has dissipated. This will indicate the persuasiveness of the deliberation or the impressionability of the participant, as well as the transience of the change in opinion that results from deliberation and reflection.

### iv. Participant compensation

All participants who participate fully in all three evenings of engagements and complete all three of the questionnaires will be given R1800 electronic vouchers as compensation. This compensation amount is based on the guidelines given by the National Health Research Ethics Council, which suggest that compensation be based on the time, inconvenience incurred and expenses [52]. Given the nature of the study, and the complexity level of the subject matter, it is expected that the participant cohort will likely comprise mostly of university students, university graduates and professionals. A further consideration is that the study will be conducted in the evenings, which will be an inconvenience for students who use the evenings to study, those with families who must attend to household duties, and those who work from home during these hours. The guidelines also recommend fair compensation for expenses, which in this study would include electricity to use their electronic devices. To fairly compensate the participants for their time and inconvenience, R300 per hour will be used as basis for calculating the compensation. (90 minutes for each of three evenings, plus 30 minutes for each of the three times the questionnaire is completed, adding up to R1 800.) In addition, participants who do not have unlimited internet access will be offered a R600 internet data voucher before the start of the study to cover their internet usage costs (if deemed necessary, on an individual basis).

## IV. Data management

### i. Data-gathering methods

All online deliberations in the plenary sessions and the sub-group sessions will be video recorded. In addition, all votes (pre-deliberation, at the deliberation, and post-deliberation) will be online and will be recorded in an online database.

### ii. Data analysis procedures

The researchers will qualitatively analyse the comments made by individual participants during the deliberations using pre-determined thematic coding. This coding will capture not only the opinions, but also changes in opinions. To avoid bias in the coding, two researchers will code the data, with a third moderating the procedure. The researchers will also, with the help of a consulting statistician, quantitatively analyse the demographic data, voting data, and where possible, quantified observations from the deliberations, in aggregate.

### iii. Data access and security

The data will be stored on the cloud in the principal investigator’s (PI) DropBox account. Only the research group members will have read-access to the relevant DropBox folder. After the conclusion of the project, the PI will remove the access for all the research team members. Only the PI knows the strong password to his DropBox account.

For the duration of the project, research group members will be allowed to download the data to their own computers. Each research team member has a strong password to access his/her computer, and will confirm this in writing to the PI. This should stop unauthorised access should such devices be stolen. After the conclusion of the project, all research group members will delete the research data from their computers and will confirm this in writing to the PI.

The data will be kept in the PI’s DropBox account for at least five years, after which the investigator may either delete it or archive it in a secure, strong password-protected cloud-based storage account similar to DropBox.

### iv. Participant protection and feedback

In the subsequent dissemination of research findings, confidentiality will be protected by anonymising the participants through code names.

After publication of the intended article(s), the PI will email each participant and provide the participant with a link to the article(s) online.

## V. Discussion

### i. Significance for practice

In view of the uncertainty and controversy surrounding novel biomedical technologies such as genome editing, it is crucial to determine public attitudes towards, and the cultural acceptability thereof, to inform future governing policies. If the public opinion about genome editing remains ignored or partially overlooked, the implementation of statutory regulations in respect thereof may result in disputes, tension and distrust between researchers, the public, government and the legislature trying to enforce governing principles. Properly informed regulations about genome editing will protect both the bodily and psychological integrity of research participants, as well as scientific and regulatory integrity, which will ultimately result in compliance with such regulations, respect and subsequent improvement of the trust relationship between the regulator and the regulated—further leading to safe and efficient navigation of novel technologies such as genome editing.

In addition, the virtual format of the study will provide valuable insights into the acceptability of the medium, engagement with research participants, and viability of serving as an alternative method for conducting research using virtual communication technologies.

### ii. Significance for policy

Every document trying to govern genome editing will have an impact on the lives of an entire population. By deliberating with the public about genome editing and ascertaining their vision, fears and concerns about it, effective policies can be drafted that address real issues affecting real lives. Such informed policies will then be able to effectively govern the symbioses between scientific development and the public’s need for advanced medical care. Considering that genome editing will affect future offspring of the genome edited individual, it is clear that genome editing poses significant and unique ethical questions. By providing detailed and thoroughly informed practical guidelines for the development of governing policies, people may be able to make fully informed decisions about genome editing and navigate their risks and preferences, while protecting themselves against exploitation, stigmatisation, discrimination and other medical or human rights abuses.

### iii. Significance for scholarship

This deliberative public engagement on genome editing research project, including the virtual format of participant engagement, will be the first of its kind in South Africa with a South African population and the aim to inform policy principles. This research project will serve as an example for future, much needed, empirical research in the legal sphere—especially research involving local populations and analysing public opinions about novel biomedical technologies. Although research designs and methodologies will depend on the particular research questions, this research design and methodology, and most importantly, the outcome thereof, will be of great value to future law students who also wish to embark on similar journeys. Further and similar research regarding novel biomedical technologies involving African populations are much needed.

### iv. Limitations

The study aims to be inclusive of a wide variety of participants, representing an array of different viewpoints. However, the design of the study is limited by the facts that the participant recruitment is only open to (1) individuals who are proficient in English as a verbal and written language; (2) individuals who have internet access; and (3) individuals who have devices capable of using Zoom. All three of these criteria will invariably exclude some groups of South Africans from participating in the deliberative public engagement, particularly non-native English speakers and those living in disadvantaged socio-economic conditions. With that being said, it should be noted that despite the small number of English native speakers in South Africa, English is the lingua franca and the language commonly used by the government in official communications with the public. Therefore, non-native English speakers in South Africa are often well versed in the language. Regarding limitations (2) and (3), there are 38.13 million active internet users in South Africa, and among them, over 36 million use mobile internet [53]. Furthermore, 24.5 million South Africans are smartphone users [54]. As such, despite the exclusions resulting from the participation criteria, involvement in the project will be accessible to over a third of South Africa’s population of approximately 58.4 million people [55]. One should also recall that the aim of the study is not to recruit a *representative* sample of the South African population (no 25–30 participants can ever be representative in a statistical sense), but rather to be *inclusive* of people that are diverse in terms of race, gender, educational attainment, age, and religion/belief. Accordingly, we suggest that the limitations of our methodology are unlikely to compromise the inclusive aim of our study, and that the results of the study, while not intended to be representative, should provide useful insights in the informed viewpoints of South Africans across the race, gender, educational attainment, age, and religion/belief spectrums.

## Supporting information

S1 Document Approval notification–expedited application. (PDF)

S2 Document Consent form: Eligibility screening to participate in virtual public deliberation on heritable genome editing. (DOCX)

S3 Document Eligibility screening. (DOCX)

S4 Document Participant resource materials: Deliberative study on heritable genome editing. (DOCX)

S5 Document Informed consent form to participate in the virtual deliberative engagement on heritable genome editing. (DOCX)

## References

[1] Lander E. S. et al. Adopt a moratorium on heritable genome editing. *Nature* 567, 165–168 (2019). doi: 10.1038/d41586-019-00726-5 30867611

[2] Macintosh K. L. Heritable Genome Editing and the Downsides of a Global Moratorium. *CRISPR J*. 2, 272–279 (2019). doi: 10.1089/crispr.2019.0016 31599680

[3] Adashi E. Y. et al. Heritable Human Genome Editing: The Public Engagement Imperative. *CRISPR J*. 3, 434–439 (2020). doi: 10.1089/crispr.2020.0049 33346718

[4] National Academy of Medicine, National Academy of Sciences & The Royal Society. Heritable Human Genome Editing. https://www.nap.edu/catalog/25665/heritable-human-genome-editing (2020) 10.17226/25665. 32897669

[5] Dryzek J. S. *Deliberative Democracy and Beyond*. (Oxford University Press, 2002).

[6] Fishkin J. S. *Democracy when the people are thinking*: *revitalizing our politics through public deliberation*. (Oxford University Press, 2018).

[7] Rawls J. *A theory of justice*. (Belknap Press of Harvard University Press, 1999).

[8] Rawls J. Reply to Alexander and Musgrave. *Q*. *J*. *Econ*. 88, 633 (1974).

[9] Rawls J. *Political liberalism*. (Columbia University Press, 2005).

[10] Rawls J. The Idea of Public Reason Revisited. *Univ*. *Chic*. *Law Rev*. 64, 765 (1997).

[11] Habermas J. & Rehg W. *Between facts and norms*: *contributions to a discourse theory of law and democracy*. (MIT Press, 2001).

[12] Cass, N. Participatory-Deliberative Engagement: a literature review. (2006).

[13] Habermas J., MacCarthy T. & Habermas J. *Reason and the rationalization of society*. (Beacon, 2007).

[14] Abelson J. et al. Deliberations about deliberative methods: issues in the design and evaluation of public participation processes. *Soc*. *Sci*. *Med*. 57, 239–251 (2003). doi: 10.1016/s0277-9536(02)00343-x 12765705

[15] Burgess M., O’Doherty K. & Secko D. Biobanking in British Columbia: discussions of the future of personalized medicine through deliberative public engagement. *Pers*. *Med*. 5, 285–296 (2008). doi: 10.2217/17410541.5.3.285 29783499

[16] Burall S. Rethink public engagement for gene editing. *Nature* 555, 438–439 (2018). 10.1038/d41586-018-03269-3 29565402

[17] Secko D. M., Burgess M. & O’Doherty K. Perspectives on Engaging the Public in the Ethics of Emerging Biotechnologies: From Salmon to Biobanks to Neuroethics. *Account*. *Res*. 15, 283–302 (2008). doi: 10.1080/08989620802388762 18972267

[18] Macnaghten P., Kearnes M. B. & Wynne B. Nanotechnology, Governance, and Public Deliberation: What Role for the Social Sciences? *Sci*. *Commun*. 27, 268–291 (2005).

[19] Pellizzoni L. Uncertainty and Participatory Democracy. *Environ*. *Values* 12, 195–224 (2003).

[20] Irwin A. Constructing the Scientific Citizen: Science and Democracy in the Biosciences. *Public Underst*. *Sci*. 10, 1–18 (2001).

[21] Jasanoff S. Science and citizenship: a new synergy. *Sci*. *Public Policy* 31, 90–94 (2004).

[22] Burgess M. M. From ‘trust us’ to participatory governance: Deliberative publics and science policy. *Public Underst*. *Sci*. 23, 48–52 (2014). doi: 10.1177/0963662512472160 24434712

[23] National Academies of Science and National Academy of Medicine. Human Genome Editing: Science, Ethics and Governance. (2017). 28796468

[24] Baylis F. ‘Broad societal consensus’ on human germline editing. *Harv*. *Health Policy Rev*. 15, 19–22.

[25] Baylis F. Human germline genome editing and broad societal consensus. *Nat*. *Hum*. *Behav*. 1, 0103 (2017).

[26] Jasanoff S., Hurlbut J. B. & Krishanu S. CRISPR Democracy: Gene Editing and the Need for Inclusive Deliberation. *Issues Sci*. *Technol*. 32, (2015).

[27] Cavaliere G., Devolder K. & Giubilini A. Regulating Genome Editing: For an Enlightened Democratic Governance. *Camb*. *Q*. *Healthc*. *Ethics* 28, 76–88 (2019). doi: 10.1017/S0963180118000403 30570466 PMC6316359

[28] Whitty J. A. et al. Harnessing the Potential to Quantify Public Preferences for Healthcare Priorities through Citizens’ Juries. *Int*. *J*. *Health Policy Manag*. 3, 57–62 (2014). doi: 10.15171/ijhpm.2014.61 25114943 PMC4122074

[29] Bartlett J., Kotrlik J. & Higgins C. Organizational Research: Determining appropriate sample size in survey research. *Inf*. *Technol*. *Learn*. *Perform*. *J*. 19, (2001).

[30] Goodin R. E. & Dryzek J. S. Deliberative Impacts: The Macro-Political Uptake of Mini-Publics. *Polit*. *Soc*. 34, 219–244 (2006).

[31] Altman D. *Citizenship and contemporary direct democracy*. (Cambridge University Press, 2019).

[32] Sunstein, C. The Law of Group Polarization. (1999).

[33] Longstaff H. & Burgess M. M. Recruiting for representation in public deliberation on the ethics of biobanks. *Public Underst*. *Sci*. 19, 212–224 (2010). doi: 10.1177/0963662508097626 20533799

[34] Horlick-Jones T., Rowe G. & Walls J. Citizen engagement processes as information systems: the role of knowledge and the concept of translation quality. *Public Underst*. *Sci*. 16, 259–278 (2007).

[35] O’Doherty K. C. & Hawkins A. Structuring Public Engagement for Effective Input in Policy Development on Human Tissue Biobanking. *Public Health Genomics* 13, 197–206 (2010). doi: 10.1159/000279621 20395688 PMC2874727

[36] Petersen A. ‘Biobanks’ “engagements”: engendering trust or engineering consent?’. *Genomics Soc*. *Policy* 3, 31 (2007).

[37] Savulescu J., Bostrom N. & Coady C. Playing God. in *Human enhancement* (eds. Savulescu J. & Bostrom N.) 155–180 (Oxford University Press, 2009).

[38] Pei D., Beier D., Levy-Lahad E. & Marchant G. Human Embryo Editing: Opportunities and Importance of Transnational Cooperation. *Cell Stem Cell* 21, 423–426 (2017). doi: 10.1016/j.stem.2017.09.010 28985523

[39] O’Doherty K. C. & Davidson H. J. Subject Positioning and Deliberative Democracy: Understanding Social Processes Underlying Deliberation: Subject Positioning and Deliberative Democracy. *J*. *Theory Soc*. *Behav*. 40, 224–245 (2010).

[40] Carusi, A. & Jirotka, M. Building Virtual Research Environments and User Engagement. (2008).

[41] O’Doherty K. C., Hawkins A. K. & Burgess M. M. Involving citizens in the ethics of biobank research: Informing institutional policy through structured public deliberation. *Soc*. *Sci*. *Med*. 75, 1604–1611 (2012). doi: 10.1016/j.socscimed.2012.06.026 22867865

[42] Constitution of South Africa. (1996).

[43] Shozi B. Something old, something new: applying reproductive rights to new reproductive technologies in South Africa. *South Afr*. *J*. *Hum*. *Rights* 36, 1–24 (2020).

[44] National Health Insurance Bill. (2019).

[45] PEW Research Centre. PEW Research Center “Public Views of Gene Editing for Babies Depend on How It Would Be Used”. (2018).

[46] World Health Organization. Global Tuberculosis Report 2015. http://www.who.int/tb/ publications/global report/en/. (2015).

[47] Deputy President Cyril Ramaphosa. World TB Day. (2016).

[48] Vanleeuw L., Mzobe Y. & Loveday M. in *District Health Barometer 2016/2017* (Health Systems Trust, 2017).

[49] Harris J. *How to be good*: *the possibility of moral enhancement*. (Oxford University Press, 2016).

[50] Heath C., Svensson M. S., Hindmarsh J., Luff P. & vom Lehn D. Configuring Awareness. *Comput*. *Support*. *Coop*. *Work CSCW* 11, 317–347 (2002).

[51] Heath C., Jirotka M., Luff P. & Hindmarsh J. Unpacking collaboration: the interactional organisation of trading in a city dealing room. *Comput*. *Support*. *Coop*. *Work CSCW* 3, 147–165 (1994).

[52] National Health Research Ethics Council. Payment of Trial Participants in South Africa: Ethical Considerations for Research Ethics Committees. (2012).

[53] Statista. Digital population in South Africa as of January 2021. https://www.statista.com/statistics/685134/south-africa-digital-population/ (2021).

[54] Statista. Number of smartphone users in South Africa from 2014 to 2023 (in millions). https://www.statista.com/statistics/488376/forecast-of-smartphone-users-in-south-africa/ (2021).

[55] Statista. Total population of South Africa in 2019, by ethnic groups. https://www.statista.com/statistics/1116076/total-population-of-south-africa-by-population-group/ (2021).

