# Supplementary material for: A virtual deliberative public engagement study on heritable genome editing among South Africans: Study protocol
Source: PLoS One. 2021 Aug 19;16(8):e0256097. doi: 10.1371/journal.pone.0256097 (PMC8376038; doi:10.1371/journal.pone.0256097)
Supplement: S2 Document — (DOCX) [file pone.0256097.s002.docx]

**Annex 1**

**Consent Form: Eligibility Screening to Participate in Virtual Public Deliberation on Heritable Genome Editing**

**Principal Investigator:** Prof Donrich Thaldar

**Responsible Institution:** University of KwaZulu-Natal

**Description of Volunteer Population:** A small collection of approximately 30 voluntary participants, residing in South Africa, will be recruited via social media from a range of different races, genders, ages, educational backgrounds, and religious affiliations. The aim is to gain the insight of individuals that are neither knowledgeable on the subject nor form part of a special interest group, and thus would not otherwise be represented. Volunteers must be willing to broaden their knowledge and understanding of heritable genome editing, and must be willing to openly share their opinions in a public deliberation process that will be virtually hosted.

**I. Purpose of the Eligibility Screening and Consent Form**

The purpose of this consent form for Eligibility Screening is to determine whether interested volunteers meet the criteria to participate in the enrolment process for the virtual deliberative engagement study. Volunteers will be informed below on the purpose and particulars of the study, and will be required to complete a questionnaire to determine their eligibility to proceed to the next stage of enrolment.

**II. Purpose of the Virtual Deliberative Engagement Study**

The purpose of the virtual deliberative engagement study on heritable genome editing in humans is for researchers at UKZN’s School of Law to determine the opinions of the public on this topic – including the legal, ethical and social aspects of heritable genome editing (how it should be regulated; how people feel about heritable genome editing; what their current understanding of this technology is; and what their concerns and hopes for this new technology are).

Heritable genome editing is a new technique developed by scientists, to remove that piece of your gene that causes disease or disability, and to replace it with a healthy piece of gene. This technique thus allows scientists to make precise **edits** to any **DNA** by altering its sequence. Heritable genome editing can be used to cure diseases and disabilities for which there was previously no cure or treatment. It can be used to enhance a person’s intelligence, memory and braveness; to enhance a person’s physical strength, speed or stamina; or to simply change a person’s eye colour, for example. These possibilities have already raised questions about who gets to decide on what heritable genome editing is or isn’t allowed, who may get access to this technique and when, and who will pay for it all.

Your participation in this study may give lawmakers insight into the public’s hopes, concerns and understanding of heritable genome editing in humans – which will enable them to better regulate this new technology. Your participation may also assist institutions better focus their education efforts, and may provide researchers with a clearer understanding of how to engage effectively and inclusively with the public on heritable genome editing.

**III. What Does your Participation Entail?**

(a) As mentioned in section I above, interested volunteers must complete the online Eligibility Screening questionnaire below, which includes reviewing a separate Informed Consent form to participate in the virtual deliberative engagement study. By completing this questionnaire, you agree for any personal data that you provide to be assessed for eligibility, and understand that in the case of non-eligibility, you will not be asked to continue with enrolment.

(b) Following the Eligibility Screening, successful candidates will be asked to study a pdf guide on heritable genome editing and watch three YouTube videos so as to deepen their knowledge on the subject. To assess their understanding, candidates will then complete an entrance exam. Upon successfully passing the entrance exam, candidates will be asked to again review the separate Informed Consent form to participate in the virtual deliberative engagement study, and should they wish to continue as a participant in the study, will be required to sign their consent to participate.

(c) Interested volunteers understand that they will not be compensated for completing the Eligibility Screening, the study of the resource material, or the entrance exam. A volunteer may at any point in the enrolment process withdraw from the screening or enrolment without any negative consequences. Your identity will be kept confidential and private by the School of Law at the University of KwaZulu-Natal.

**IV. Duration**

The duration of the enrolment process is detailed below. Please note that once the Eligibility Screening is successfully completed, candidates will have until a specified date that will be communicated to them to complete the entrance exam.

3.1 Eligibility screening questionnaire and brief review of Informed Consent form: 10–15 minutes.

3.2 Reading through the resource material and watching the videos: own pace, but approx. 2–3 hours; all three videos are approx. 15 minutes in total.

3.3. Entrance Exam: 45 minutes.

3.4. In-depth review and completion of the Informed Consent form to participate in the virtual deliberative engagement study: 30 minutes, depending on the familiarity with the research objectives and concepts.

**V. Eligibility Screening**

(a) Following your consent to the Eligibility Screening, you will be provided with a questionnaire of approximately 10 questions. All questions must be answered, and your personal details (including name, age and contact details) will be requested.

(b) The deadline for completing the Eligibility screening is the [DATE].

(c) Upon completion of the questionnaire, you will be notified as to whether you are: (1) eligible to continue, (2) waitlisted, or (3) rejected. In the case of (2), volunteers may be waitlisted for reasons including ambiguous or unforeseen responses, or the number of successful volunteers exceeding the study limits. Those who are waitlisted will be informed no later than [DATE] if their status changes.

**VI. Study materials and Entrance Exam**

(a) If you are eligible to continue, you will be given access to the resource material and three YouTube videos.

(a) After the materials have been studied, you may access the entrance exam online. You will be asked to answer 20 multiple choice questions.

(b) **All** exam questions must be answered correctly to successfully continue on to the informed consent to participate in the study.

(c) The entrance exam may be taken as many times as necessary to achieve correct responses to all questions.

(d) The final attempt for the entrance exam must be undertaken on or before the [DATE].

**VII. Review of Informed Consent for the Participation in the Virtual Deliberative Engagement Study**

(a) Upon successfully passing the entrance exam, you will be asked to review the Informed Consent form for participation in the virtual deliberative engagement study. You should carefully read and understand the procedures, risks and discomforts of participation, and should you have any questions, contact the researchers (details provided below).

(b) Should you wish to proceed as a participant in this study, you will be required to sign the online form electronically. Should you not wish to proceed as a participant, you would not complete the form.

(c) The study will be conducted on the [DATE].

**VIII. Confidentiality during the Eligibility Screening**

If you choose to submit the results of your Eligibility Screening, as described above, your name or identity will be linked to your responses and shared **only** with the researchers involved in this study. The researchers will take reasonable precautions to ensure that your name and your Eligibility Screening responses and results are kept confidential, and will not sell your personal information to any institution, company or person for financial gain or commercial profit. Any data that are publicly shared for the purposes of research will be anonymised.

**IX. Benefits, Risks and Discomforts with the Eligibility Screening**

(a) Benefits of participating in Eligibility Screening include the potential opportunity to participate in the deliberative study, which would inform researchers and policymakers on public opinion regarding heritable genome editing.

(b) Risks and discomforts: there are no apparent risks or discomforts in participating in the Eligibility Screening. None of the questions elicit responses that could any in manner be used to incite prejudice or bias against the candidate.

**X. Refusal or Withdrawal of Participation**

(a) Participation in this Eligibility Screening and enrolment process is voluntary. You do not have to participate in the Eligibility Screening or enrolment process, and you may withdraw your participation and request that the researchers delete any personal data that you may have submitted, at any time.

(b) The researchers may decide, at their sole discretion, to end your participation in this Eligibility Screening or enrolment process at any time, without any compensation for your time.

(c) If you choose to submit the results of your Eligibility Screening, regardless of whether you are (1) eligible to continue, (2) waitlisted or (3) rejected, the researchers may retain certain information about you, including your name and the date of submission of your Eligibility Screening results. The researchers will delete all other data supplied by you within 6 months of your withdrawal or rejection from this Eligibility Screening.

**XI. Research-Related Contact Information**

If you have any further questions or concerns related to the Eligibility Screening, the enrolment process, or the deliberative engagement study, you may contact Prof Donrich Thaldar at [ThaldarD@ukzn.ac.za](mailto:ThaldarD@ukzn.ac.za).

**XII. Informed Consent for Eligibility Screening**

I ___________________________________________________ (Full names and Surname)

hereby confirm that:

- I fully understand the contents of this document and the nature, risks and benefits of the Eligibility Screening.
- I understand that by signing my name and surname below on this date, that I freely and voluntarily consent to undertake the Eligibility Screening.
- I understand that inaccurately representing my full name on this form will disqualify me from continuing with the Eligibility Screening, and that I may not sign on behalf of another person even I am their parent, guardian or hold a power of attorney or comparable authority with respect to such a person.
- I have been given an opportunity to ask questions about the procedures related to the study and I was given answers to my satisfaction.
- I understand that I may withdraw from this study at any time, and will only be compensated for my time upon successfully passing the entrance exam.
- I may contact the researcher, Prof Donrich Thaldar, at [ThaldarD@ukzn.ac.za](mailto:ThaldarD@ukzn.ac.za) if I have any further questions.
- If I am concerned about an aspect of this study or about the researcher then I may contact the University of KwaZulu-Natal’s Humanities and Social Sciences Research Ethics Committee via the Committee’s administrator, Ms Mariette Snyman at HssrecLms@ukzn.ac.za or 031-260-8350/4609.

SIGNATURE OF PARTICIPANT___________________________DATE _______________
