# Supplementary material for: A virtual deliberative public engagement study on heritable genome editing among South Africans: Study protocol
Source: PLoS One. 2021 Aug 19;16(8):e0256097. doi: 10.1371/journal.pone.0256097 (PMC8376038; doi:10.1371/journal.pone.0256097)
Supplement: S3 Document — (DOCX) [file pone.0256097.s003.docx]

**Annex 2**

**ELIGIBILITY SCREENING**

| **Question** | **Response** |
| --- | --- |
| 1. Nationality | - South African - Other |
| 2. Gender | - Female - Male - Other |
| 3. Race | - Asian - Black - Coloured - Indian - White - Other |
| 4. Highest level of Education | - Primary School (grades 1–7) - High School - Diploma - Bachelor degree - Honours degree - Masters degree - PhD - Other |
| 5. Proficiency of English as a written and spoken language | - Yes - No |
| 6. Age |  |
| 7. Religion/belief system (you may select more than one option) | - Agnostic - African Traditional - Atheist - Buddhist - Christian - Humanist - Hindu - Jewish - Muslim - Other |
| 11. Are you willing to learn more about genome editing and the ethical debates surrounding it? | - Yes - No |
| 12. Are you willing to form your own opinions on the various uses of genome editing and to discuss your opinions with others? | - Yes - No |
| 13. Are you willing to participate in three evenings of online meetings that will be recorded for research purposes? | - Yes - No |
| 14. Do you have sufficient Internet access and proficiency with, and access to, the Zoom platform? (data to be provided by the study). | - Yes - No |
| **Contact number:**  **Contact e-mail:**  **Alternative contact number:** |  |
