# Supplementary material for: A virtual deliberative public engagement study on heritable genome editing among South Africans: Study protocol
Source: PLoS One. 2021 Aug 19;16(8):e0256097. doi: 10.1371/journal.pone.0256097 (PMC8376038; doi:10.1371/journal.pone.0256097)
Supplement: S4 Document — (DOCX) [file pone.0256097.s004.docx]

**PARTICIPANT RESOURCE MATERIALS: DELIBERATIVE STUDY ON HERITABLE GENOME EDITING**

We live in a time where science and technology have transformed many areas of our lives –for better or worse. But people rarely get an opportunity to think about how new technology will affect them and the world before that technology is put on the market.

This is one of those rare opportunities, where we, as researchers, would like to find out what you think about a certain new technology that might affect your life in the future. In this document, we first explain what our deliberative study is about. Then, we will help you to better understand the scientific and ethical issues surrounding the new technology called ‘heritable genome editing’. The goal of this study material is to give you the information you need to help you have an informed discussion about this potentially world-changing technology.

# 1. What is our deliberative study about?

The purpose of our study is to understand the opinions of a diverse group of South Africans on the topic of ‘heritable genome editing’. We understand that there might be many different views, and we would like to hear yours. This means that you will engage with other participants in an open and democratic way, by listening to the opinions of others and sharing your own. Where possible, we would hope to find common ground on some of the thinking, but it is not a necessary outcome. Some people may choose to change their opinions as we progress through the study when they hear the views of others, or when they hear your views, and that is acceptable. In fact, we encourage everyone to approach the deliberations with an open mind and try to find common ground.

The deliberative study will take the form of three Zoom meetings, each lasting about 90 minutes. These Zoom meetings will be scheduled for three consecutive weekday evenings, after working hours. All these meetings will be video recorded for research use only.

To guide the deliberations, we will ask the participants several questions, and ask the participants to give their opinions. The participants will be asked to answer these questions on their own on three occasions: (1) a week before the deliberations, (2) directly after the deliberations, and (3) one week after the deliberations.

## i. How do I qualify to be a participant?

To qualify to participate in our study, you must successfully complete an online entrance exam. This will show that you have read this document, watched the three videos, and that you are familiar with their content. The entrance exam consists of 15 multiple choice questions. To successfully complete this, you must provide correct answers to **all** the questions. You can retake the entrance exam as many times as you like. We provide a link to the online entrance exam at the end of this document.

The entrance exam will remain open until the research team decide to close it – which will likely be once we have enough candidates that are sufficiently diverse.

You may leave the study at any time without any negative consequences. Please inform one of the researchers if you would like to leave the study. Any information you have provided up to that point will be kept by the researchers, but your connection to the research data will not be disclosed.

## ii. Confidentiality

Participants’ identities will be known to each other, and to the researchers involved. However, all involved will agree not to disclose the identities of the participants. The data collected during our study – including the video recordings of the Zoom meetings – will be analysed by the researchers. The results of the study will be published as academic articles by the researchers, and will also be presented at conferences and workshops. Your identity and that of the other participants will never be disclosed. We will strictly adhere to the provisions of the Protection of Personal Information Act (POPIA).

## iii. Compensation

It is important that participants will be able to use Zoom’s video function to see each other during the meetings. Since the Zoom meetings using video will require internet data, we will offer each participant a R600 data voucher for the network of their choice (if necessary) before the Zoom meetings take place.

Also, participants will be compensated with a R1200 electronic voucher for their time in terms of participating in the three Zoom meetings and answering the three sets of policy questions. This voucher will be given to participants on completion of the project.

## iv. Ethics is important

Ethics approval for this study has been given by the University of KwaZulu-Natal’s Humanities and Social Sciences Research Ethics Committee. Any questions can be emailed to the Committee’s administrator, Ms Mariette Snyman – HssrecLms@ukzn.ac.za. You can also call her on 031-260-8350/4609.

You are also welcome to contact the principal investigator of the study, Prof Donrich Thaldar – ThaldarD@ukzn.ac.za.

# 2. Introduction to genome editing

To help you understand heritable genome editing, we now answer the questions:

- 1. What is genome editing?
  2. What is CRISPR-Cas9?
  3. Why edit human genes?
  4. What are the risks?
  5. Should we ban, allow, limit or support heritable genome editing?

2.6 What kinds of changes should be allowed?

# 2.1. What is genome editing?

We first need to ensure that you understand basic genetics concepts, such as ‘DNA’ and ‘genes’, before we explain ‘genome editing’. Please watch these three videos on YouTube – each video is about 5 minutes long:

- [This video explains DNA](https://www.youtube.com/watch?v=zwibgNGe4aY).
- [This video explains genes](https://www.youtube.com/watch?v=5MQdXjRPHmQ).
- [This video explains genome editing.](https://www.youtube.com/watch?v=I5_2c52OPFw&t=84s)

As you saw in the last video, genome editing is also called ‘gene editing’. The word ‘genome’ refers to the complete set of an organism’s DNA, whereas a gene is one part of that DNA. A simple comparison is that a gene is like a sweet, and the genome, the entire bag of sweets. Although ‘genome editing’ and ‘gene editing’ are often used to mean the same thing, we use only the term ‘genome editing’.

Genes and DNA are contained in all the cells of the body. Cells can be broadly grouped into the reproductive cells (sperm and egg cells) and all the other cells that are not reproductive cells. Other cells that are not reproductive cells make up most of a person, e.g. skin cells, liver cells, and blood cells. When genome editing occurs in adults, these are the cells whose genes are edited. This limits the changes in the genes to a particular part of the body, e.g. the liver. To make sure that the gene edits make a difference, many cells would have to be edited. For example, the liver is made up of millions of cells. If the liver is diseased, editing the genes of only 10 cells at one point would not be helpful. Batches of hundreds of cells would need their genes to be edited, possibly at regular intervals, in order for the liver to heal.

Reproductive cells (sperm and egg cells) are what come together from a female and male during sexual reproduction to eventually form a new human organism. The male’s sperm goes into the female’s egg to form one cell that then divides constantly to form new cells. This early stage of the human organism is called an embryo, and when it is born, it will be a baby. So any genes in the sperm or egg cells will be in the baby/embryo. If you change (edit) these genes in these reproductive cells or in the embryo itself when it is only a few cells, this will affect all the cells in that eventual person’s body. In this way, these changes to the genes in the baby are permanent.

So, in an adult’s cells that are not reproductive cells, genome editing will only affect that adult individual. The edited genes will not pass down to any offspring (child), and so are not inherited. However, if you edit the genes of the reproductive cells or the early embryo, these changes will be passed down to the offspring. This kind of genome editing is *heritable*, that is, it can be inherited. This study focuses only on heritable genome editing.

# 2.2. What is CRISPR-Cas9?

New technology is making the possibility of heritable genome editing a reality. This technology is called CRISPR-Cas9, which can be used to make precise changes at specific points in a genome. Think of piece of ribbon that you want to cut at a precise point. CRISPR-Cas9 is like the scissors that allow you to make those cuts accurately. Then you can add or remove pieces more easily.

CRISPR-Cas9 allows the scientist to either insert new gene sequences, or, to simply delete genes at a specific position in the genome. This is why it has sometimes been described as a genetic ‘cut-and-paste’ tool.

# 2.3. What are the benefits to editing human genes?

Many medical conditions are caused by mutations in human genes. These mutations can occur naturally (without a scientist or geneticist interfering with your genes directly), and they often happen in the body without you knowing. Genome editing may be a way to cure these conditions. Unfortunately, up until recently, finding an effective and safe way to make changes to genes was difficult.

CRISPR-Cas9 has shown a great deal of promise, as it is relatively cheap and seems to be able to change human genes with precision. Previous ‘cutting’ technologies were not as precise, which meant that there was a higher chance of error. CRISPR-Cas9 has greatly reduced that risk of error, but is still being refined. However, it is not yet safe enough for use by the general public. There is excitement about it reaching a point where this is possible, as human genome editing might give us a way to treat devastating genetic diseases such as Huntington’s disease, Tay Sachs disease, and some forms of cancer. With heritable genome editing, the changes that are made to genes can be passed down to future generations. So, rather than treating genetic diseases, we could prevent them.

The potential uses of CRISPR-Cas9 are not limited to genetic diseases. We could also change certain traits of future children that are controlled by genes, including eye colour and height.

# 2.4. What are the risks?

With all this potential for good, there also comes a lot of risk. Firstly, the fact that CRISPR-Cas9 is a relatively cheap and easy method of genome editing, means that it is open to abuse. We saw this in 2018, when a Chinese scientist unlawfully altered two human embryos that were then implanted in a woman. Although the children were successfully born, this caused controversy for two reasons. One, the Chinese scientist did not follow the law or proper research ethics, and up till now we still do not fully know what scientific activities were performed. Two, scientists widely agree that the technology was – and still is – not yet ready for use in humans. The changes the scientist tried to make may also have had unintended effects, because CRISPR-Cas9 has been reported to sometimes make alterations to genes other than the ones it was meant to make changes to.

Another risk is that because the technology is so easy to use, and is not always strictly controlled, individuals can use CRISPR-Cas9 for their own, ‘DIY’ activities. This opens up scientific research and the possibility of discoveries, but can also be dangerous if the person does not know what they are doing, or if they have harmful intentions.

In addition to these technical challenges, genes work together in complex ways to control how our cells function. While a few parts or functions of our bodies are controlled mostly by a single gene, most are a product of the relationship between several genes. Scientists have yet to fully understand how some of these relationships work with each other. Therefore, editing a single gene, which we think is responsible for one thing (like eye colour), may affect something else (like nail growth).

Like any other technology that is to be used in humans – for example a new medicine – scientists must first improve CRISPR-Cas9 to ensure that it is highly reliable. This is done in cells grown in the lab. Then they must test it in various animal models to ensure it works. After this, they can conduct clinical trials in humans before it can be made generally available. Scientists are still far away from clinical trials for heritable genome editing. However, it is possible that these clinical trials will take place in the future, and that heritable genome editing might be proven to be safe and effective.

This possibility raises ethical questions. In the following paragraphs, we will try to provide a balanced overview of the main ethical arguments for and against allowing heritable genome editing. This overview was not written to persuade you one way or the other, but rather to inform you of both sides of the debate, and to stimulate your own thoughts.

# 2.5 Should we ban, allow, limit or support heritable genome editing?

CRISPR-Cas9 is a technology of great potential, but also risks. This has led to heated debate about whether it would be ethical to use this technology even if it is proven to be safe and effective, or whether the law should prohibit it, allow it, limit it, or promote it. The big question at the centre of the debate is whether parents should be allowed to choose to have children with changed or edited genes. Is making changes to the human genome that will be passed to future generations, a line that should not to be crossed?

There are a wide range of views in this area both for and against heritable genome editing. We will highlight some of the main arguments here, and provide a summary of these arguments at the end of this section (*Table 1*):

## i. Should we use germline genome editing to prevent disease?

The most obvious argument in favour of allowing heritable genome editing is that its widespread use presents the possibility of preventing a serious genetic disorder in a new-born, and reducing the chance that this disease could be passed on to future generations of that family. If made available to the general public, heritable genome editing may lead to a world where some people do not suffer from devastating diseases caused by mutations in a gene, such as muscular dystrophy.

But not all genetic diseases are caused by a single gene; some are caused by mutations in several genes, and the relationship between genes and disease is not well understood. For example, there is strong evidence to suggest that there is a genetic component to developing schizophrenia (children of people with schizophrenia are almost twice as likely to develop the illness). But we have not as yet discovered a singular ‘schizophrenia gene’, and people with a family history of the disorder may never develop it. However, in the future, scientists may find the genes that cause diseases like schizophrenia. In such cases, heritable genome editing would potentially provide a way to prevent these genetic diseases, or at least lower the number of people who develop them.

Some critics argue that heritable genome editing, whatever the reason for it, is wrong, and that we should rather rely on conventional medicine. Their reasons will be discussed in the paragraphs below. Other critics argue that once we allow any kind of heritable genome editing of embryos, it will put us on a ‘slippery slope’. In their argument, the selection of genes in a child will not just be to lower risk of disease, but also to enhance or change features such as intelligence, hair colour, and athleticism, which are not regarded as necessarily life-changing for a better quality of life, so leading to ‘designer babies’.

## ii. Is genome editing is ‘unnatural’ or ‘playing God’?

One common reason for opposing the use of heritable genome editing, is that it is seen as ‘unnatural’ or as ‘playing God’. People believe that human genes should always be determined the ‘natural way’. They believe it is wrong for humans to choose whether a future child will have particular genes. Some people’s religious beliefs are also that one cannot change human genes. They view this technology as humans stepping into the domain of God, by determining (through genes) who a person will be. In other words, heritable genome editing will give too much power to us humans, and we should prevent further development of it.

Opponents of these arguments say that new technologies are often viewed as unnatural at first. Yet, over time, they are accepted. Another point is that we cannot say that using technology to determine a person’s genetic makeup is wrong, as it is only one of many ways parents try to influence their future child’s characteristics. For example, people commonly choose specific partners based on their appearance and intelligence, in the hopes of passing these characteristics on to their children. This is widely viewed as acceptable, so why should using heritable genome editing to do this be viewed any differently? One possible answer is that heritable genome editing goes further than partner selection. Can humans be trusted with such power over the genes of future generations? Should we then forbid further developing heritable genome editing? Humanity has many powerful technologies that we try to manage for the good. Is this possible with heritable genome editing?

Others argue that practising heritable genome editing is not unnatural at all, but is very natural to humans. Altering the natural world for our own good is something which humans have done for thousands of years – whether this is by using cell-phones to communicate, or using medicines to counter diseases. Editing the human gene is, for those in favour of it, just another way for humans to use their minds to change nature to improve their lives as humans.

## iii. Should parents be free to choose?

Heritable genome editing – if proven to be safe, effective and useful – will be the latest technology that can be used to allow people to make choices about if, when, and how they want to have children. Some have been critical of the possibility of heritable genome editing being used in human reproduction ,because of the concerns about whether it is morally acceptable. These critics argue that there is no good reason for parents to resort to genome editing given that there are other alternatives such as adoption and preimplantation genetic testing (PGT). PGT refers to the process of genetically testing embryos for particular genes, then using only those embryos with the desired genes to create a child and disposing of the rest.

However, for many people, other alternatives to having children may not work. For example, they may not be eligible to adopt, or it may be very important to them to have a child that is genetically related to them. For prospective parents who have a serious genetic disease that is likely to be passed on to their children, heritable genome editing would open up a new avenue for them by allowing them to have a child that is genetically related to them. In the case of parents who both carry a gene for a disease, genome editing may be the only way for them to have a child genetically related to both of them, and which is disease free. And even if just one parent carries the genes for a disease, a couple may still prefer genome editing over other alternatives like PGT, because it would not require the destruction of embryos. For some people, this would be justified because of deeply held religious or moral convictions against the unnecessary destruction of human life.

Should prospective parents need any reason at all to justify using heritable genome editing? Even if there are alternatives, in democracies like South Africa where freedom is respected, it is presumed that people have the right to do what they wish in their private lives, which includes using novel technologies. This applies even if some people may not approve of that technology or how it is used. Therefore, those in favour of heritable genome editing argue that the law cannot ban the practice if it is proven to be safe, because doing so would undermine the freedom, choice, and reproductive rights of future parents. For these people, heritable genome editing is a deeply personal choice that should be left to the individual. They feel that others should not take away this choice, nor decide when it is appropriate to make it because of their own opinions on human genome editing.

Another argument on freedom of choice is whether, by choosing for their unborn children, parents are taking away the freedom of choice from these children. In other words, by ‘choosing’ the child’s genes, the parent makes a life defining choice for their future child, and does so without their consent. In response, one might ask: how does this differ from parents choosing surgery, vaccinations or medications for their children? In reality, parents are not only allowed but expected to make choices on their child’s behalf, without the child’s consent, and often these choices may influence not only the child but several future generations.

## iv. Is heritable genome editing ‘eugenics’?

Eugenics is a set of beliefs and practices that aim to improve the genetic quality of a human population according to what one group believes to be an improvement. Historically, in the early 20th century, eugenics programmes in Britain, much of Europe, the USA and Nazi Germany were characterised by demeaning and negatively treating people and groups judged to be ‘inferior’, and promoting or favouring those judged to be ‘superior’. For example, Nazi Germany favoured the ‘blue eyes, blond hair’ look of the Aryan race, and looked down harshly on the characteristics of the Jewish race. Many of the eugenics programmes included human rights abuses such as forced sterilisations, mass murders and genocides. It is argued by some that because heritable genome editing means choosing certain genes for their future children over other genes, parents will be practising a form of eugenics.

Those in favour of heritable genome editing respond to this argument by saying that it is unfair to compare heritable genome editing with past eugenics programmes. What made the historic eugenics programmes wrong was that they took away the freedom and choice of people. These programmes forced onto all people in a country a particular vision of what ‘better’ humans would be like. Heritable genome editing, on the other hand, when made freely available, and subject to individual choice, would not be like this. Instead, people would be given the freedom to choose whether to use this technology or not. It will promote the freedom of those with genetic diseases by giving them a choice to have children that are genetically related to them – while not passing on their genetic disease.

## v. Will genome editing promote discrimination against people with disabilities?

Most of us agree that people living with disabilities are entitled to the same care and respect as any other person. But what does this mean? Does this mean we use genome editing to help them improve their quality of life? Or does genome editing not respect their different way of life?

If parents make use of heritable genome editing, they will be choosing certain genes for future generations. This means that parents will be choosing one kind of person (the person with edited genes) over another kind of person (the person with unedited genes). For example, if parents use heritable genome editing to edit (or ‘correct’) the genetic mutations that cause autism to ensure that their child is born without autism, they are effectively choosing a child without autism over a child with autism. Is this ethical? Is this not a form of discrimination against people living with disabilities? Will this make society less tolerant or accepting of differences amongst humans – which includes disabilities?

The typical response to these questions is that it is important to acknowledge that it is not the same thing to say that disability comes with difficulty and hardship and therefore ought to be avoided, than it is to say that disabled persons are less valuable than abled persons. It is also different when avoiding disability before a child’s birth through therapeutic intervention, compared to discriminating against an actual living person with a disability. When we fasten our seatbelts in a car, we are trying to avoid becoming disabled in the event of a car crash. Is this unethical? Fastening one’s seatbelt is not the same as treating someone with a disability in a disrespectful manner. Respect for disabled persons should also be created by the policies and educational measures we adopt. These policies and educational measures should promote tolerance of difference and disability. Therefore, even if disability is prevented by genome editing in some and not others, those others will not necessarily be discriminated against.

## vi. Is there a ‘right’ to an ‘unaltered genome’?

Some are concerned that heritable genome editing will harm future persons, by taking away their ‘right’ to an ‘unaltered genome’ (the complete set of genes). They argue that each of us is entitled to be born with a genome that is the product of the natural processes of procreation — not a genome that was chosen by our parents. They also argue that it is wrong for parents to choose the genetic characteristics of their future child, because it affects the child’s ‘right’ to an ‘open future’. Parents would be determining a part of the child’s identity and future. Not only is this seen as unfair towards the child, but the child born with edited genes may also be psychologically harmed by knowing he/she was created in this way.

Defenders of heritable genome editing reply that arguments such as these overemphasise the role of genes in our characteristics and identity. It is well established that individuals are a product of not only their genes (nature), but also of their environment (nurture). So it cannot be said that by selecting for certain genes, the entirety of one’s identity is chosen, and that the person concerned is not free to create a future for themselves as they see fit. Furthermore, the concern that children will be psychologically harmed by the knowledge that they are genetically modified is, at this point, pure speculation. What we do know is that studies with children born by non-traditional means, such as donor-conceived and surrogacy children, indicate that they have the same psychological well-being as other children.

There is also the question of what is ‘unaltered’ or ‘unchanged’? In the natural world, genes change all the time, and are called mutations. Some mutations are good, and some are harmful. But without genome editing, these are still occurring, and can occur at any point in one’s life.

## vii. Will heritable genome editing lead to increased inequality?

Concerns have been raised that genetic technologies will worsen existing inequalities in society. This might lead to the emergence of a genetically ‘enhanced’ superior class that would have an unfair advantage over those who could not afford access to these technologies. The risk of this is greater in countries like South Africa because of the wide income gap and the difficulty in accessing healthcare by some members of society.

Some argue, however, that the use of genetic technologies may never become widespread enough to have such a social impact. Given the current state of technology, heritable genome editing will be possible only if done together with in vitro fertilisation (IVF), and not together with natural conception (having sexual intercourse). Given that most people do not need to use IVF to conceive, it would automatically limit the application of heritable genome editing. But, what if technology improves to such an extent that heritable genome editing can somehow be done together with natural conception? Say, for instance, an early embryo can be gene edited by the pregnant mother simply swallowing a special tablet? Those who support heritable genome editing say that the correct approach would be for society to take measures to make heritable genome editing as widely available as possible – rather than restricting it. This might prevent inequality and promote human development.

*Table 1: Arguments against and for allowing heritable genome editing in humans*

| AGAINST: Manipulating the human genome is wrong, and will lead to ‘designer babies’. | FOR: Genome editing is good because it presents the possibility of preventing serious genetic disorders. |
| --- | --- |
| AGAINST: Genome editing is ‘unnatural’, and choosing your child’s genetic characteristics is ‘playing God’, something which parents should not have the power to do. | FOR: New technologies are often viewed as ‘unnatural’ at first, but become accepted over time. Influencing the genetic characteristics of a child is not new or unique to genome editing. |
| AGAINST: There is no good reason for parents to resort to genome editing given that there are other alternatives such as adoption and PGT. | FOR: For many people, other alternatives to having children may not work, and parents may have reasons for preferring genome editing over the alternatives. |
| AGAINST: Parents using genome editing means they are choosing certain genes for their future children over other genes, and this is eugenics. | FOR: Genome editing is different from past eugenics programmes that took away the freedom and choice of people. Genome editing would be subject to individual choice. |
| AGAINST: If parents use genome editing to edit the genetic mutations that cause disability, they are effectively choosing a child without disability over a child with disability. This sends the message that people with disability are less valuable and is a form of discrimination. | FOR: Acknowledging that disability ought to be avoided is not the same thing as saying disabled persons themselves are undesirable. It is different to avoid disability before a child’s birth through therapeutic intervention, than to discriminate against an actual living person with a disability. |
| AGAINST: By choosing to use genome editing, parents are taking away their child’s ‘right’ to an ‘unaltered genome’. It is wrong for parents to choose the genetic characteristics of their future child because it affects the child’s ‘right’ to an ‘open future’. | FOR: Alterations in the genome are natural and common. It cannot be said that by selecting for certain genes, the entirety of one’s identity is chosen for them. People remain free to create a future for themselves as they see fit. |
| AGAINST: Genetic technologies will worsen existing inequalities in society. This might lead to the emergence of a genetically ‘enhanced’ superior class that would have an unfair advantage over those who could not afford access to these technologies. | FOR: The use of genetic technologies may never become widespread enough to have such a drastic social impact. And if it does, the correct approach would be for society to take measures to make genome editing as widely available as possible, rather than restricting it. |

# 2.6. What kinds of changes should be allowed?

Even if we accept that genome editing is not necessarily wrong and may be permitted, there is still significant disagreement over what *kind* of gene edits should be allowed. Here we give a general overview of the main arguments on this issue. The arguments for what kind of changes should be allowed falls into two broad categories: therapy vs enhancement, and serious vs non-serious diseases.

## i. Therapy vs enhancement

Some people draw a line between therapy and enhancement. They argue that heritable genome editing should only be allowed to treat diseases or disabilities that can be inherited. Their reason is that this has a ‘therapeutic’ purpose. They believe that heritable genome editing for non-therapeutic purposes or enhancement should not be acceptable. An example of an enhancement would be the selection for higher intelligence, or a desired skin tone.

We can ask: Why is genetic enhancement wrong? If using heritable genome editing for therapeutic purposes is found acceptable, why not also allow heritable genome editing for enhancement? After all, parents are allowed to invest as much time and energy in their children as they choose, thereby potentially giving them an added advantage over other children whose parents do not choose to or cannot do so. For instance, parents can teach their children to read and write before they go to school to give them an educational advantage. Once their children are at school, some parents can also afford to send their children for extra mathematics classes or to private schools to give them a competitive advantage. These actions are not viewed negatively. As we enhance children in other ways, would it then be wrong of parents to *genetically* enhance their future children?

Also, the line between therapy and enhancement is not always clear. Our understanding of what it means to be ‘normal’ and/or to deviate from the ‘normal’ – to have a disease, a disability or a disorder (be it physiological, psychological, or otherwise) – depends on the society and environment we live in. Ideas of disease and disability change over time and between places, and depend on a particular legal, political, social, and cultural context. Some may see previously unheard of conditions as ‘problems’ in society today, which need to be changed. For example, so-called ‘toxic masculinity’ (the pressures for men to behave in a certain way that causes harm to others and themselves) was not in the past identified as a problem that needed to be changed, but is being called out now. Where previously there was no way of genetically addressing these issues, in the future there might be. This might mean, with the example of toxic masculinity, that one could use heritable genome editing to ensure that a future boy will be less competitive and more cooperative, which then could be seen as a type of ‘therapy’.

We must also remember that homosexuality was seen as a psychological disorder (with the implication that it needed therapy) until relatively recently. Accordingly, it has been argued that the therapy/enhancement distinction is not always reliable or useful as a means of separating what should be allowed, from what should not be allowed. A further point is that if heritable genome editing is allowed at all, each specific gene edit should be considered on its own benefits and risks.

## ii. Serious vs non-serious

Another argument is that genome editing should only be used for ‘serious’ diseases. Some argue that because genome editing carries greater risk than natural reproduction, it should only be used in exceptional, serious cases. In this case, ‘serious’ acts as a starting point for us to start talking about genome editing in humans as a possibility. But what happens when genome editing is proven safe and effective, and that risk is reduced? Do we then allow less serious cases to be treated?

Then there is the difficulty in determining what is ‘serious’. One person’s experience of a disease or condition may differ from another’s for many reasons. For example, a person with sensitivity to sunlight who works indoors may find the condition far more manageable than someone who works outdoors. We also do not necessarily know what may become a more serious threat over time. For example, malaria is a serious disease in Mozambique, but not in most parts of South Africa. Over time, with climate change, however, it may become a much more serious threat in South Africa.

A further point, even if we can define what is ‘serious’, is whether we should limit genome editing to this at all? Would this then not prevent us from getting access to the highest standard of health possible for people, which is right in most countries?

# 3. Conclusion

You now know a little bit more about what heritable genome editing is and what it can do. We have demonstrated that there are many, often conflicting, views and ideas on how and if heritable genome editing should be allowed.

We would like to hear your opinions. If you would like to participate in our study, the next step is to complete the online entrance exam. Here is a link to it:….
