# Supplementary material for: A virtual deliberative public engagement study on heritable genome editing among South Africans: Study protocol
Source: PLoS One. 2021 Aug 19;16(8):e0256097. doi: 10.1371/journal.pone.0256097 (PMC8376038; doi:10.1371/journal.pone.0256097)
Supplement: S5 Document — (DOCX) [file pone.0256097.s005.docx]

**Annex 3**

**Informed Consent Form to Participate in the Virtual Deliberative Engagement on Heritable Genome Editing**

**Principal Investigator:** Prof Donrich Thaldar

**Responsible Institution:** University of KwaZulu-Natal

**Description of Volunteer Population:** A small collection of approximately 30 voluntary participants, residing in South Africa, will be recruited from a range of different races, genders, ages, educational backgrounds, and religious affiliations. The aim is to gain the insight of individuals that are neither knowledgeable on the subject nor form part of a special interest group, and thus would not otherwise be represented. Volunteers must be willing to broaden their knowledge and understanding of genetic editing, and must be willing to openly share their opinions in a public deliberation process that will be virtually hosted.

**I. Purpose of the Virtual Deliberative Engagement Study**

The purpose of the virtual deliberative engagement study on heritable genome editing is for the researcher at UKZN’s School of Law to determine the opinions of the public on this topic – including the legal, ethical and social aspects of heritable genome editing in humans (how it should be regulated; how people feel about heritable genome editing; what their current understanding of this technology is; and what their concerns and hopes for this new technology are).

Heritable genome editing is a new technique, developed by scientists, to remove that piece of your gene that causes disease or disability, and replace it with a healthy piece of gene. This technique thus allows scientists to make precise **edits** to any **DNA** by altering its sequence. Heritable genome editing can be used to cure diseases and disabilities for which there was previously no cure or treatment. It could potentially be used to enhance a person’s intelligence, memory and braveness; to enhance a person’s physical strength, speed or stamina; or to simply change a person’s eye colour, for example. These possibilities have already raised questions about who gets to decide on what heritable genome editing is or isn’t allowed, who may get access to this technique and when, and who will pay for it all.

Your participation in this study may give lawmakers insight into the public’s hopes, concerns and understanding of heritable genome editing, which will enable them to better regulate this new technology. Your participation may also assist institutions better focus their education efforts, and may provide researchers with a clearer understanding of how to engage effectively and inclusively with the public on heritable genome editing.

**II. Review of Informed Consent for the Participation in the Virtual Deliberative Engagement Study**

(a) Upon successfully passing the entrance exam, you will be asked to review the Informed Consent form for participation in the virtual deliberative engagement study. This process will take approximately 30 minutes. You should carefully read and understand the procedures, risks and discomforts of participation, and should you have any questions, contact the researchers (details provided below).

(b) Should you wish to proceed as a participant in this study, you will be required to sign the online form electronically. Should you not wish to proceed as a participant, you would not complete the form.

**III. What does your participation in the Virtual Deliberative Engagement Study Entail?**

(a) A participant in the study will be required to provide their opinion on heritable genome editing in humans during a virtual discussion on the Zoom platform. You will be provided with a R600 data voucher for your network service provider to cover the Internet usage costs.

(b) A total of 30 participants will be selected for the deliberative study, which will be held on three weekday evenings [DATES]. Participants, by agreeing to be a part of this study, agree to attend and actively engage in all the sessions, which are anticipated to last about 1.5 hours each.

(c) A week before the engagement is due, all participants will be provided with access to a website with the policy questions that will be deliberated on at the engagement. Each participant will be requested to consider all the policy questions and indicate his or her answer, at that (pre-deliberation) stage.

(d) At the first evening, all participants and facilitators will introduce themselves. The facilitator will establish the ground rules, such as that participants should endeavour to limit their speaking turns to 30 seconds at the longest. After this, the agenda will be introduced to the participants. In particular, the participants will be provided with the policy questions that they will have to deliberate on that evening. Each evening will have a theme:

- Evening 1: prevention of heritable genetic conditions
- Evening 2: editing for immunity
- Evening 3: editing for enhancement

Following these sessions, the participants will be randomly divided into six sub-groups for smaller break-away meetings. These sub-groups will each be facilitated by law academics and postgraduate students. Each sub-group will have an initial 20 minutes to deliberate on the policy proposals entailed by the evening’s theme. You will be asked to consider some of the discussed values and to suggest how you think these values should be balanced with other values and rights, and most importantly what your personal opinion about these issues are. You may ask the facilitator to better explain heritable genome editing technology and its consequences to you, to make sure that you fully understand.

This will be followed by a whole group deliberation of 20 minutes.

(e) If consensus eludes the deliberation at this point, the process of breaking up into sub-groups and returning for a whole group meeting will be repeated. Note that the sub-groups will not remain the same, but will be randomly re-composed for the second round of break-away deliberations. Similarly, the sub-groups will be randomly re-composed every evening. The aim is to attempt to avoid that the members of a sub-group become entrenched in their sub-group position.

Throughout deliberations consensus will not be forced. Large group discussions on each issue will be concluded with a final vote on the recommendation(s). The vote will serve two purposes: 1) to provide closure to discussion on an issue and allow the facilitator to move the discussion forward; and 2) to ensure that minority views are not missed and are clearly documented.

(f) The study will be structured around policy proposals related to heritable genome editing corresponding with the themes of the three evenings.

(g) Post-deliberation – a week after the engagement is done, each participant will again be requested to answer the policy questions that were deliberated on at the engagement, on his or her own. The purpose will be to investigate whether individual participants changed their mind at the deliberation, and whether they may again change their minds after the deliberation has ended.

(h) A participant who completes **all** three evenings by attending and fully engaging the discussion will receive a R1800 voucher (electronically) for their time.

**IV. Data management**

(a) Data-gathering methods

All online deliberations in the whole group sessions and the sub-group sessions will be video recorded. In addition, all votes (pre-deliberation, at the deliberation, and post-deliberation) will be online and will be recorded in an online database.

| I specifically consent to the group sessions and the sub-group sessions will be video recorded for purpose of this research study. | |
| --- | --- |
| YES | NO |

(b) Data analysis procedures

The researchers will qualitatively analyse the comments made by individual participants during the deliberations. The researchers will also quantitatively analyse the demographic data, voting data, and where possible, quantified observations from the deliberations, in aggregate.

(c) Data access and security

The data will be stored on the cloud in the PI’s DropBox account. Only the research group members will have read-access to the relevant DropBox folder. After the conclusion of the project, the PI will remove access for all the research team members. Only the PI knows the strong password to his DropBox account.

For the duration of the project, research group members will be allowed to download the data to their own computers. Each research team member has a strong password to access his/her computer, and will confirm this in writing to the PI. After the conclusion of the project, all research group members will delete the research data from their computers and will confirm this in writing to the PI.

The data will be kept in the PI’s DropBox account for at least five years, after which the investigator may delete it or archive it in a secure, strong password-protected cloud-based storage account similar to DropBox.

(d) Participant protection and feedback

After publication of the intended article(s), the PI will email each participant and provide the participant with a link to the article(s) online.

**V. Confidentiality during the study**

If you choose to participate in the research study, as described above, you agree to keep the identity of the other research participants confidential. It will be made clear to all participants and researchers that the identities and any identifiers of participants may not be shared with anyone outside the research study.

For the purposes of collating and analysing the raw data, your name or identity will be linked to your responses and shared **only** with the researchers involved in this study. Raw data will not be shared with anyone outside of this study.

Any refined data that is publicly shared for the purposes of research will be anonymised by assigning the participants with code names.

The researchers will take reasonable precautions to ensure that your name and your responses are kept confidential, and will not sell your personal information to any institution, company or person for financial gain or commercial profit.

**VI. Benefits, Risks and Discomforts of the Virtual Deliberative Engagement Study**

(a) Benefits of participating in the virtual deliberative engagement study include the potential opportunity to inform researchers and policymakers on public opinion regarding heritable genome editing.

(b) Risks and discomforts: there are no apparent risks in participating in the study. None of the questions elicit responses that could any in manner be used to incite prejudice or bias against the participant. While the facilitators of the sessions will make the utmost attempt to create a conducive and inviting environment for the sharing of opinions, participants should be aware that fellow participants may have strong, opposing/conflicting opinions, and that this should not be seen as a personal attack on the participant’s own opinions or belief systems.

**VII. Refusal or Withdrawal of Participation**

(a) Participation in this study is voluntary. You do not have to participate in the study, and you may withdraw your participation, and request that the researchers delete any personal data that you may have submitted, at any time.

(b) The researchers may decide, at their sole discretion, to end your participation in this study. If their reasons are based on a participant’s improper conduct (misinformation, refusal to engage constructively), the researchers retain the right to withhold further compensation.

**VIII. Research-Related Contact Information**

If you have any further questions or concerns related to the virtual deliberative engagement study, you may contact Prof Donrich Thaldar at [ThaldarD@ukzn.ac.za](mailto:ThaldarD@ukzn.ac.za).

**IX. Informed Consent for Virtual Deliberative Engagement study**

I ___________________________________________________ (Full names and Surname)

hereby confirm that:

- I fully understand the contents of this document and the nature, risks and benefits of the virtual deliberative engagement study.
- I understand that by signing my name and surname below on this date, that I freely and voluntarily consent to undertake the virtual deliberative engagement study.
- I understand that inaccurately representing my full name on this form will disqualify me from continuing with the virtual deliberative engagement study, and that I may not sign on behalf or another person, even I am their parent, guardian or hold a power of attorney or comparable authority with respect to such a person.
- I have been given an opportunity to ask questions about the procedures related to the study and I was given answers to my satisfaction.
- I understand that I may withdraw from this study at any time, and will only be compensated for successfully passing the entrance exam and separately for attending and actively engaging in all three deliberative sessions.
- I may contact the researcher, Prof Donrich Thaldar at [ThaldarD@ukzn.ac.za](mailto:ThaldarD@ukzn.ac.za) if I have any further questions.
- If I am concerned about an aspect of this study or about the researcher then I may contact the University of KwaZulu-Natal’s Humanities and Social Sciences Research Ethics Committee via the Committee’s administrator, Ms Mariette Snyman at HssrecLms@ukzn.ac.za or 031-260-8350/4609.

SIGNATURE OF PARTICIPANT___________________________DATE _______________
