# Supplementary material for: A virtual deliberative public engagement study on heritable genome editing among South Africans: Study protocol
Source: PLoS One. 2021 Aug 19;16(8):e0256097. doi: 10.1371/journal.pone.0256097 (PMC8376038; doi:10.1371/journal.pone.0256097)
Supplement: S1 Document — (PDF) [file pone.0256097.s001.pdf]

UNIVERSITY OF KWAZULU-NATAL
INYUVESI YAKWAZULU-NATALI

29 March 2021

Prof Donrich Willem Thaldar (59413)
School Of Law
Howard College

Dear Prof Thaldar,

Protocol reference number: HSSREC/00002595/2021
Project title: A virtual deliberative public engagement study on heritable genome editing among South Africans
Non-Degree

Approval Notification – Expedited Application

This letter serves to notify you that your application received on 12 March 2021 in connection with the above, was reviewed by the Humanities and Social Sciences Research Ethics Committee (HSSREC) and the protocol has been granted **FULL APPROVAL**.

Any alteration/s to the approved research protocol i.e. Questionnaire/Interview Schedule, Informed Consent Form, Title of the Project, Location of the Study, Research Approach and Methods must be reviewed and approved through the amendment/modification prior to its implementation. In case you have further queries, please quote the above reference number. PLEASE NOTE: Research data should be securely stored in the discipline/department for a period of 5 years.

This approval is valid until 29 March 2022.
To ensure uninterrupted approval of this study beyond the approval expiry date, a progress report must be submitted to the Research Office on the appropriate form 2 - 3 months before the expiry date. A close-out report to be submitted when study is finished.

All research conducted during the COVID-19 period must adhere to the national and UKZN guidelines.

HSSREC is registered with the South African National Research Ethics Council (REC-040414-040).

Yours sincerely,

Professor Dipane Hlalele (Chair)

/dd

Humanities and Social Sciences Research Ethics Committee
Postal Address: Private Bag X54001, Durban, 4000, South Africa
Telephone: +27 (0)31 260 8350/4557/3587 **Email:** hssrec@ukzn.ac.za **Website:** http://research.ukzn.ac.za/Research-Ethics

Founding Campuses: Edgewood Howard College Medical School Pietermaritzburg Westville

INSPIRING GREATNESS
